# Mitochondrial Molecular Pathophysiology of Nonalcoholic Fatty Liver Disease: A Proteomics Approach

**DOI:** 10.3390/ijms17030281

**Published:** 2016-03-15

**Authors:** Natalia Nuño-Lámbarri, Varenka J. Barbero-Becerra, Misael Uribe, Norberto C. Chávez-Tapia

**Affiliations:** 1 Traslational Research Unit, Médica Sur Clinic & Foundation, Mexico City 14050, Mexico; nnunol@medicasur.org.mx (N.N.-L.); vbarberob@medicasur.org.mx (V.J.B.-B.); 2 Obesity and Digestive Diseases Unit, Médica Sur Clinic & Foundation, Mexico City 14050, Mexico; muribe@medicasur.org.mx

**Keywords:** proteomics, NAFLD, mitochondrial dysfunction

## Abstract

Nonalcoholic fatty liver disease (NAFLD) is a chronic liver condition that can progress to nonalcoholic steatohepatitis, cirrhosis and cancer. It is considered an emerging health problem due to malnourishment or a high-fat diet (HFD) intake, which is observed worldwide. It is well known that the hepatocytes’ apoptosis phenomenon is one of the most important features of NAFLD. Thus, this review focuses on revealing, through a proteomics approach, the complex network of protein interactions that promote fibrosis, liver cell stress, and apoptosis. According to different types of *in vitro* and murine models, it has been found that oxidative/nitrative protein stress leads to mitochondrial dysfunction, which plays a major role in stimulating NAFLD damage. Human studies have revealed the importance of novel biomarkers, such as retinol-binding protein 4, lumican, transgelin 2 and hemoglobin, which have a significant role in the disease. The post-genome era has brought proteomics technology, which allows the determination of molecular pathogenesis in NAFLD. This has led to the search for biomarkers which improve early diagnosis and optimal treatment and which may effectively prevent fatal consequences such as cirrhosis or cancer.

## 1. Introduction

Non-alcoholic fatty liver disease (NAFLD) is a clinicopathological condition that is commonly associated with dyslipidemia, insulin resistance, cardiovascular disease, obesity metabolic syndrome and type 2 diabetes mellitus (T2DM) [1]. Moreover, the liver is targeted by signals from other tissues, including adipose tissue, the gut and its microbiota [2], comprising a wide spectrum of liver damage, ranging from simple steatosis to steatohepatitis [3], which is a major health problem affecting an estimated 25% of the adult population worldwide. Although NAFLD is highly prevalent on all continents, the highest prevalence rates were reported in South America (31%) and the Middle East (32%) while the lowest prevalence was reported in Africa (14%). Also, the prevalence between the United States and Europe is similar, and an interesting finding was the relatively high prevalence found in the Asian population (27%) [4].

NAFLD can progress to nonalcoholic steatohepatitis (NASH) in 12%–40% of cases. NASH can be distinguished by the presence of hepatocyte ballooning, apoptosis, inflammatory infiltrates, and collagen deposition. Over a period of 10–15 years, 15% of patients with NASH will exhibit progression to liver cirrhosis. Annually, 4% of hepatic decompensation is generated by cirrhosis that has not been caused by viral hepatitis, while the overall risk of generating cancer in 10 years is 10% [5].

Currently, proteomics are an essential approach that have improved the study of the complex pathogenesis of NAFLD, becoming more outstanding since they have been applied in the health sciences and industry [6] and being useful in the determination of pathophysiology and identifying new markers for disease diagnosis [7].

Proteomics provide essential information of the biologically active entity named protein, which includes its post-translational modifications and interactions with other proteins [8]. Proteomic techniques are primarily based on electrophoresis and mass spectrometry [9]. In recent years, genomics, proteomics, and bioinformatic techniques have been developed synergistically and have experienced a surprising development, which has brought about major advances in medicine.

### 1.1. Identification of Specific Proteins through in Vitro Studies

*In vitro* models are necessary for elucidating the mechanisms of liver damage in NAFLD, as they are for understanding the complex network of cellular interactions, apoptosis and oxidative stress, the mechanisms that lead to mitochondrial damage which promotes fibrosis.

Hepatic oxidative stress and injury are mechanisms associated with polyploidy, which is one of the most dramatic changes that can occur in the genome [10]. A hepatocyte NAFLD model has shown that oxidative stress triggers the activation of a G2/M DNA damage checkpoint, preventing the activation of the cyclin B1/CDK1 complex, which causes an inefficient progression through the S/G2 phases, suggesting that polyploidy in mononuclear cell populations is an early event in NAFLD development [11].

During liver injury, perpetuation of the insult induces progressive deterioration of hepatic damage with the production of extracellular matrix (ECM) remodeling components, which contribute to uncontrolled ECM turnover [12], leading to an excessive accumulation of extracellular proteins, proteoglycans, and carbohydrates that ends in a pathological state that is called fibrosis [13]. Components of the fibrotic liver ECM had been previously cataloged by sodium dodecyl sulfate polyacrylamide gel electrophoresis (SDS–PAGE) separation and mass spectrometry (GeLC-MS)–based proteomics approaches [14]. An *in vitro* liver fibrosis model using mass spectrometry analysis in cell-derived ECM identified 61 structural or secreted ECM proteins (48 proteins for a hepatic stellate cell line, LX-2, and 31 proteins for human foreskin fibroblasts) [14]. Several proteins identified in this study have been linked with fibrotic processes that occur in the liver and other organs; fibrillin, which was previously implicated in the activation of transforming growth factor β (TGF-β) storage, was among those proteins [15]. Furthermore, two new fibrotic constituent proteins identified in this study, CYR61 and Wnt-5a, were also validated in the fibrotic liver [14]. GELC-MS–based proteomics coupled to an ECM-enrichment strategy in an *in vitro* model of liver fibrosis may be a valuable tool for determining the mechanisms underlying fibrosis and for the identification of novel therapeutic targets or biomarkers.

Fibrosis is not the only mechanism of liver damage; apoptosis has also been studied since it is one of the most important features of NAFLD [16]. The participation of certain proteins, such as cytochrome b5, annexin A5 and A6, and protein disulfide isomerase fragments, has been confirmed in murine and human cell apoptosis models [17]. On the other hand, it has been reported that cholesterol induces Bax and caspase-3 [18], which may be important proteins for apoptosis; however, cholesterol did not increase the expression of p53 and Bcl-2 in steatotic cells, suggesting an important role for cell death mechanisms in hepatocytes [19].

Furthermore, proteomic techniques could be useful in other scenarios such as liver regeneration; an analysis was performed through label-free quantitative mass spectrometry in which human embryonic stem cells were differentiated into hepatocyte-like cells to investigate the effects of the cell secretome, which demonstrated that hepatocyte-like cells derived from stem cells contribute to the recovery from injured liver tissue in mice by delivering trophic factors that support liver regeneration [20].

The application of this strategy to different *in vitro* disease models may therefore significantly improve identifying specific proteins, and provide the first step toward elucidating the mechanisms which underlie fibrosis and novel therapeutic targets or biomarkers.

### 1.2. In Vivo NAFLD Studies

**Figure 1:**
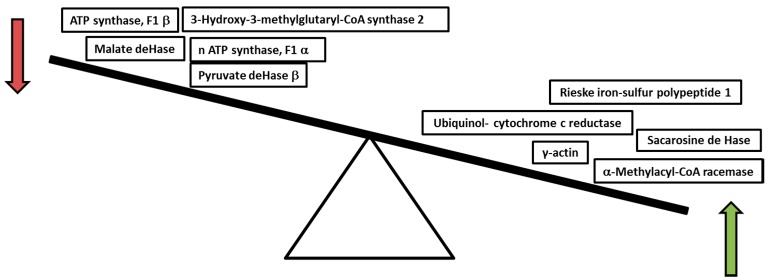
Mitochondrial proteins altered by high-fat diet.

Obesity is related to several diseases, such as NAFLD and NASH, being linked to mitochondrial dysfunction and deficiency of nitric oxide (NO). Chronic consumption of a high-fat diet (HFD) in a murine model induces NASH, and it is accompanied by profound changes in mitochondrial bioenergetics. Conversely, HFD decreased the activity of cytochrome c oxidase and increased sensitivity to the NO-dependent inhibition of mitochondrial respiration [21]. According to HFD intake, a densitometry analysis revealed that 22 proteins were significantly altered, whereas 67 proteins remained unchanged. The last events are a bit far from proposing a mechanism; however, this response could be considered as a regulatory mechanism according to the microenvironment where it develops (Figure 1) [21].

Chronic exposure of mice to a HFD induces hepatic steatosis, modifying the liver mitochondrial proteome, including changes in proteins related to oxidative phosphorylation, protein folding, and lipid and sulfur amino acid metabolism [21]. Mitochondrial dysfunction may be generated by high concentrations of reactive oxygen species (ROS) which inhibit the respiratory chain and integrity of mitochondrial DNA and also contribute to organelle toxicity, the suppression of fatty acid oxidation and the rise in lipid peroxidation [22].

Liver steatosis may be due to an excess of fatty acids (FA), glucose, lipotoxicity, or insulin resistance (IR), and it induces *de novo* lipid synthesis by the activation of nuclear receptors such as sterol regulatory element-binding protein 1 (SREBP-1), carbohydrate-responsive element-binding protein (ChREBP-1), and peroxisome proliferator-activated receptor γ (PPARγ) [23]. Moreover, PPARγ activation increased cellular free FA uptake, exceeding the adaptive pathways of hepatic lipid export and catabolism, suggesting an adipogenic transformation of hepatocytes [24]. The presence of steatosis is tightly associated with chronic hepatic inflammation, an effect mediated in part by activation of the Ikκ-b/NF-κB signaling pathway.

A murine model of steatosis induced with a HFD increases NF-κB activity, which is associated with the elevated hepatic expression of pro-inflammatory cytokines such as TNF-α and IL-1 which are activated by ROS created by lipid peroxidation, responsible not only for promoting insulin resistance and Kupffer cell activation, but also for mediating cholesterol and triglyceride metabolism [25,26].

TNF-α act upon leukocyte infiltration in the liver, contributing to intracellular oxidative stress and mitochondrial dysfunction; in fact, TNF receptor adaptor proteins initiate the phosphorylation of mitogen-activated protein kinases (MAPK 1), which in turn activate c-Jun N-terminal kinases (JNK) [27]. Prolonged activation of the downstream signaling molecule JNK was found to promote inflammation and apoptosis [28], amplifying hepatocyte damage [29].

Studies in JNK2 knockout mice indicated that this protein might be important for caspase 8 activation and apoptosis mitochondrial pathways in response to TNF-α [30]. Treatment with anti-TNF-α antibodies improved mitochondrial respiration and inflammation, and alleviated hepatic steatosis in mouse models of NASH [31]. Also, it has been seen that Gegenqinlian decoction (GGQLD), a Chinese herbal medicine, can decrease serum elevated TNF-α levels, being an optimal approach for managing lipid metabolic, inflammatory, and histological abnormalities via the PPARγ/TNF-α pathway in NAFLD [26].

Mitochondria adjust to lipid accumulation in hepatocytes raising the levels of β-oxidation; nevertheless, increased substrate transfer to the mitochondrial electron transport chain leads to a rise in ROS production and finally insulin resistance, playing an important role in hepatic lipid metabolism [32]. In a murine model study, with the use of gel electrophoresis (DIGE) and MALDI-TOF techniques, 95 proteins were identified to exhibit significant changes during the development of NAFLD, whereas protein down-regulation was observed for enoyl coenzyme A hydratase (ECHS1), which catalyzes the second step of the mitochondrial β-oxidation of fatty acids, probably because of HFD-related hepatic steatosis [33]. These findings suggest an important role for ECHS1 in lipid accumulation in *in vivo* NAFLD models [34].

Furthermore, the HFD-mediated decrease in ATP synthase subunits (F1α and β) may also compromise mitochondrial energy conservation; these findings, together with a decrease in the content of malate and pyruvate dehydrogenase, which are key mitochondrial metabolism enzymes, provide strong evidence supporting the occurrence of bioenergetics dysfunction in response to chronic exposure to a HFD, which can be linked to NAFLD liver proteome changes [35]. Moreover, some proteins associated with acetyl-CoA intake and oxidative stress are molecular markers of hepatic steatosis in ob/ob mice that have been identified by liver mitochondrial 2D-DIGE proteomics [36]. Also, a comparative study of liver mitochondrial proteomics, using Ingenuity Pathway Analysis software (IPA; Ingenuity Systems, Mountain View, Redwood City, CA, USA), found that among the 1100 protein analyzed, aldehyde dehydrogenase 2 (ALDH2), and 3-hydroxy-3-methylglutaryl-CoA synthase 2 (HMGCS2) were altered [37]. In summary, analysis of sub-mitochondrial and cellular proteomes indicates that metabolic adaptations occurring in hypertriglyceridemic mice hepatocytes induce an enhanced acetyl-CoA, glycerol-3-phosphate, ATP and Nicotinamide adenine dinucleotide phosphate (NADPH) availability for *de novo* triglyceride (TG) biosynthesis. They also strongly suggest that the cytosol of HuApoC-III mouse hepatocytes is the subject of an important oxidative stress, probably as a result of free fatty acid (FFA) over-accumulation, iron overload and enhanced activity of some ROS-producing catabolic enzymes [38].

Also, the increase of intracellular triacylglycerols may be promoted by the inhibition of lipoprotein assembly and secretion [23]. Recently it has been found that fetuin A is an adaptor protein for saturated fatty acid–induced activation of Toll-like receptor 4 signaling, promoting lipid-induced insulin resistance; also, fetuin B secretion from the liver is increased by steatosis and diminishes glucose lowering through insulin-independent mechanisms [39].

It is important to study alcoholic fatty liver disease (AFLD) since it shares some hepatocyte injury mechanisms with NAFLD. AFLD appears in 90% of people who consume ≥60 mg per day of alcohol; however, both have the deterioration of mitochondrial functions because of protein nitration in common [40]. Under normal conditions, these function capacity alterations can be managed by properly using the antioxidant host defense system and by the removal of nitrated proteins, which can serve as a defense mechanism against nitroxidative stress–related harmful consequences [35].

Peroxynitrite and protein nitration were suggested to be the main causes of acute and chronic AFLD injury models [41]. Also, several mouse models have been used to evaluate the effect of protein nitration on nitroxidative stress [42]. For instance, the role of protein nitration has been studied in mouse strains with ablated genes that are involved in the regulation of superoxide and NO levels [43] in which the identification of peptides that originate from nitrated proteins can be performed using matrix-assisted laser desorption/ionization time-of-flight mass spectrometry (MALDI-TOF MS) [44,45].

Moreover, knockout inducible nitric oxide synthase (iNOS) mice with a Lieber–De Carli ethanol liquid diet exhibit a markedly decreased level of nitrated proteins, which confers resistance to AFLD and, together with protein nitration, inhibits complex I (NADH ubiquinone oxidoreductase) and complex V (ATP synthase) activities in models of acute and chronic alcohol exposure [46,47].

The authors suggest that these damaging effects are probably caused by protein nitration, as the administration of iNOS inhibitors and peroxynitrite scavengers, such as uric acid, ameliorated the ethanol-induced nitration and the inhibition of activity and mitochondrial depletion of ATP synthase. In addition, the deletion of superoxide dismutase 2 (SOD2) would scavenge superoxide and block peroxynitrite formation, yielding the extension of mitochondrial DNA depletion, whereas SOD2 over-expression yielded opposite outcomes [47].

On the other hand, cytosolic SOD1 also exhibits a protective role against ethanol-mediated hepatic damage [48]. In SOD1-deficient mice, the levels of protective hepatic ATP content and SOD2 expression were decreased, whereas oxidative damage and nitro-Tyr formation were elevated in response to ethanol feeding, thus leading to greater hepatic injury [41]. Up to this point, evidence suggests that hepatic mitochondria from ethanol-fed murine models are more sensitive to NO and reactive nitrogen species. It seems that after ethanol exposure, mitochondrial liver dysfunction might develop a cytosolic antioxidant defense, which could be an important feature of chronic hepatotoxicity damaging the proteome and genome [49].

In regards to the inflammatory response, it is important to mention that ethanol hepatotoxicity was significantly prevented through a mechanism that involves a decrease in tumor necrosis factor α (TNF-α) formation, in hepatocytes isolated from alcohol-fed rats, through the SDS–PAGE technique [50]. It was not surprising that TNF-α knockout mice exhibited a significantly less severe ethanol-mediated hepatotoxicity, markedly accompanied by lower levels of protein Tyr nitration [51].

The Fernandez-Checa group have shown that mitochondrial free cholesterol loading in steatohepatitis sensitizes to TNF and Fas through mitochondrial glutathione (GSH) depletion [31]. Protein Tyr nitration and its functional consequences might explain the role of protein nitration in promoting many forms of liver disease, including AFLD and NAFLD [52]. The levels of protein nitration are correlated with the increased levels of hepatic transaminases, steatosis, and necrosis [43]. It is also very important to study the NO bioavailability throughout the course of NAFLD. In an HFD mouse model, it was shown that NO contents were initially increased, causing mitochondrial damage accompanied by alterations in mitochondrial proteins, such as thiolase, complex I (NADH ubiquinone oxidoreductase), aldehyde dehydrogenase 2 (ALDH2), and complex V (ATP synthase); in contrast, NO levels decreased at later stages of NAFLD [43]. NO might be an encouraging inflammatory regulatory marker according to the NAFLD damage stage.

### 1.3. Human Studies

Based on the hypothesis that liver injury in NAFLD and NASH is caused by protein effectors, as described for the *in vitro* and *in vivo* models, human studies are critical because they may help establish biomarkers that can be used for an earlier diagnosis and more effective treatments.

Dr. Feldstein’s group reported that extracellular vesicle (EV) proteomes carry a selective antigenic composition that might be used to diagnose NAFLD non-invasively. They analyzed cell death, inflammation, and antioxidant and pathological angiogenesis in steatotic mice, finding that some functional activities of oxidoreductase, hydrolase, endopeptidase inhibitors, signal transducers and lipid binding proteins were abundantly expressed in EVs [53]. Another study in patients with simple steatosis showed that a group of cytochrome P450 family proteins, such as CYP2E1, CYP4A11, and CYP2C9, are upregulated, being associated with lipid droplets (LDs). On the other hand, mitochondrial proteins were found to be downregulated, suggesting that these enzymes are involved in NAFLD development and mitochondrial dysfunction. Increased adipose differentiation-related protein (ADRP) and fatty acid synthase (FAS) mRNA and protein expression were found to be upregulated in the LD fractions of patients with steatosis. It has been recently recognized that in fatty liver disease, the LD-associated protein 17β-HSD13 expression was upregulated [54].

There are several molecules that have been associated with liver damage progression. For instance, two important proteomic studies in adult patients using liver tissue and serum respectively, with and without NAFLD, revealed an increased expression of lumican (a keratan sulphate proteoglycan involved in collagen cross-linking and epithelial–mesenchymal transition) [55]. The expression of lumican was similarly abundant in obese patients with normal liver histology and in obese patients with simple steatosis; however, it was over-expressed in mild progressive NASH patients [56]. Thus, lumican is expressed differentially across the progressive stages of NAFLD, and not just in patients with moderate to advanced fibrosis, raising the possibility of over-expressed hepatic lumican as an early marker of a profibrotic state in patients with NAFLD [57]. Also, fatty acid-binding protein 1 (FABP-1) is another protein involved in multiple biological functions, such as intracellular fatty acid transport, cholesterol and phospholipid metabolism, which plays an important facilitative role in hepatic fatty acid oxidation [58,59]. FABP-1 is relatively over-expressed in patients with simple steatosis compared with those with obesity; however, throughout the NAFLD stages, it was observed that FABP-1 was significantly under-expressed in patients with mild and progressive NASH [60].

A novel analysis of hepatic peptides performed on an electrospray ionization mass spectrometry (ESI-MS) biosystem (an analytical technique that can provide both qualitative (structure) and quantitative (molecular mass or concentration) information on analyte molecules after their conversion to ions) [61] was conducted on several phenotypes of fatty liver disease, where 1362 hepatic proteins were assessed. Several proteins were consistently abundant among study groups, whereas albumin, hemoglobinβ, hemoglobinα, dihydropyrimidinase, enolase, the metal-transport protein ATX1, and HSP gp96 were likely differentially abundant because of the biological effects of increased hepatic lipid content or inflammation [56]. Furthermore, it has been observed that serum and hepatic TNF-α levels are elevated in patients with NAFLD, correlating with the animal models which had already been studied. Conversely, inhibition of TNF-α signaling improves insulin resistance (IR) and histological parameters of NAFLD [26].

In another study which involved NAFLD patients who underwent bariatric surgery, quantified protein peak intensity levels were selected from SELDI-TOF mass spectrometry [62]; the results revealed that fibrinogen γ was elevated, playing a role in blood clotting and serving as a depot for active fibroblast growth factor receptor 2 (FGF2) in the blood, and it may be connected to liver fibrosis [63]. However, the role of fibrinogen γ in NAFLD remains speculative and needs to be well defined. Moreover, this study involves patients with varying stages of NAFLD. Several protein biomarkers were identified and classified from priority 1 to 4, according to quality identification (ID); priority 1 proteins have the greatest likelihood of correct ID (multiple unique sequences identified), such as transgelin 2, retinol-binding protein 4, lumican, and paraoxonase 1, among others [62].

Importantly, it seems that each protein may have biological significance in the microenvironment in which it is expressed. For instance, the fibrinogen β chain, retinol-binding protein 4 (RBP4), serum amyloid P component, lumican, transgelin 2, and CD5 antigen-like exhibit differential levels of expression among patient groups and present a global success rate of 76%, whereas complement component C7, the insulin-like growth factor acid labile subunit, and transgelin 2 present a global success rate of 90% wherein they are characterized by simple steatosis and NASH and are able to accurately differentiate between control subjects and patients with all forms of NAFLD [62]. RBP4 is an important protein synthesized by the liver and adipose tissue, carrying vitamin A in the blood; it has been involved in the development of IR and has been related to increased NAFLD severity [64].

NAFLD development has been associated with elevated serum hemoglobin levels, being independent of body mass index, type 2 diabetes, and other metabolic diseases [29,65]. One of the potential explanations for the observed associations between increased hemoglobin and NAFLD may be related to oxidative stress, catalyzed by iron excess accumulation and probably causing thrombosis, leading to hepatocyte injury [66,67]. The relationship between serum hemoglobin and NAFLD may be partially modulated by haptoglobin levels, which act as an antioxidant binding to free hemoglobin and inhibiting the hemoglobin-induced oxidative damage [65]. Furthermore, excessive erythrocytosis increased hemoglobin in NAFLD subjects without a diagnosis of metabolic syndrome (MS), and this should be considered in the selection of cases for histological assessment of disease severity and progression [68]. On the other hand, Lixin Zhu *et al.* showed that in NASH, hemoglobin is highly expressed and synthesized in hepatocytes, being released into the circulatory system and providing a possible explanation for serum free hemoglobin [69]. Therefore, hemoglobin measurements should be considered part of the clinical evaluation markers for severity of liver damage in patients with NAFLD [67,70].

Finding clinical biomarkers that have arisen from proteomic technologies, which reveal biological reactions and could distinguish NAFLD from NASH, is of great importance (Table 1). However, accurate human studies which involved protein analysis related to mitochondrial dysfunction are lacking. Oxidative-nitrated stress proteins play a major role in stimulating damage in various hepatic diseases, including AFLD and NAFLD mediated by ethanol. As these proteins are essential for normal mitochondrial function, protein nitration might lead to irreversible modification of the respiratory-chain proteins [29].

## 2. Conclusions

**Figure 2:**
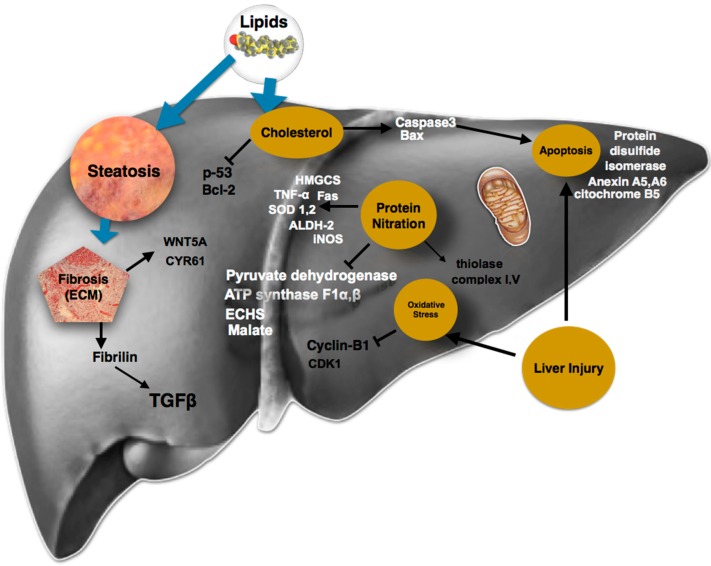
Activation and inhibition of different proteins in NAFLD.

*In vitro* studies are the basis for elucidating the pathogenic network that is involved in NAFLD, which is interesting because of the recognition of some proteins involved in liver fibrosis. Conversely, *in vivo* studies have focused on the bioenergetics dysfunction caused by chronic exposure to HFD, which can be linked to changes in protein interactions in the liver proteome between NAFLD and NASH (Figure 2) [14]. Human studies have revealed the importance of novel proteins that were identified as having a high rate of confidence in the presence of NAFLD and NASH and seem to emerge as good marker candidates (Table 1). Deeper and more accurate human studies will be required to identify the network of complex proteomes that underlies the pathogenesis related to mitochondrial dysfunction, where its functional consequences might explain the pathophysiological mechanism which follows many forms of liver diseases.

## Figures and Tables

**Table 1:** Proteins involved in NAFLD and potential markers for NAFLD.

Research Context	Protein	Implications and Findings	Study	Year
**Cell Cycle**	Cyclin B1 CDK1	Polyploidy in mononuclear cell populations is an early event in NAFLD development.	Gentric, Maillet *et al.* [11]	2015
**Fibrosis**	Fibrillin TGF-β CYR61 Wnt-5a Lumican Fibrinogenγ FGF2	Mechanisms underlying fibrotic processes. Early marker of a profibrotic state in patients with NAFLD.	Lorena, Darby *et al.* [15]	2004
			Rashid, Humphries *et al.* [14]	2012
			Fitzpatrick and Dhawan [57]	2014
			Younossi, Baranova *et al.* [63]	2005
**Apoptosis**	Cytochrome b5 Annexin A5 Annexin A6 Bax Caspase 3 Caspase 8	Important role for cell death mechanisms in hepatocytes.	Jayaraman, Roberts *et al.* [17]	2005
			Yamaguchi, Chen *et al.* [18]	2004
			Zhu, Xie *et al.* [19]	2014
			Sabapathy, Hochedlinger *et al.* [30]	2004
**Lipid synthesis**	SREBP-1 ChREBP-1 PPARγ Acetyl-CoA Glycerol-3-phosphate Fetuin A Fetuin B	Adaptive pathways of hepatic lipid export and catabolism. Hepatocytes are subjected to an important oxidative stress. Promote lipid-induced insulin resistance.	Anderson and Borlak [23]	2008
			Al Sharif, Alov *et al.* [24]	2014
			Ehx, Gerin *et al.* [38]	2014
**Inflammation**	NF-κB TNF-α IL-1 MAPK1 JNK	Promote insulin resistance, Kupffer cells activation, cholesterol and triglyceride metabolism, intracellular oxidative stress.	Cai, Yuan *et al.* [25]	2005
			Wang, Liu *et al.* [26]	2015
			Lim, Dillon *et al.* [29]	2014
**β-Oxidation**	ECHS1	Lipid accumulation in NAFLD.	Zhang, Yang *et al.* [33]	2010
			Lewis, Hagstrom *et al.* [34]	2002
**Oxidative stress**	ALDH2 HMGCS2 Hemoglobin Haptoglobin	Acetyl-CoA consumption and oxidative stress as molecular markers of hepatic steatosis. Catalyze the accumulation of iron in excess.	Douette, Navet *et al.* [36]	2005
			Peinado, Diaz-Ruiz *et al.* [37]	2014
**Antioxidants**	SOD2 SOD1	Protective role in mitochondrial DNA depletion, and hepatic ATP content.	Mansouri, Tarhuni *et al.* [47]	2010
			Kessova, *et al.* [48]	2003
**Lipid droplets**	CYP2E1 CYP4A11 CYP2C9	Enzymes involved in mitochondrial dysfunction and the development of NAFLD.	Su, Wang *et al.* [54]	2014
**Lipid metabolism**	FABP-1	Intracellular fatty acid transport, cholesterol and phospholipid metabolism, and plays an important facilitative role in hepatic fatty acid oxidation.	Binas and Erol Higuchi [58]	2007
			Kato *et al.* [59]	2011

Cyclin-dependent kinase 1 (CDK1), Transforming growth factor beta (TGFβ), Cysteine-rich angiogenic inducer 61 (CYR61), Wingless-Type MMTV Integration Site Family, Member 5A (Wnt-5a), Fibroblast Growth Factor 2 (FGF2), BCL2-Associated X Protein (Bax), Sterol regulatory element-binding protein 1 (SREBP-1), Carbohydrate-responsive element-binding protein 1 (ChREBP-1), Peroxisome proliferator-activated receptor gamma (PPARγ), Nuclear factor κB (NF-κB), Tumor necrosis factor α (TNF-α), Interleukin 1 (IL-1), Mitogen-activated protein kinase 1 (MAPK1), c-Jun N-terminal kinase (JNK), Enoyl-CoA hydratase short chain 1 (ECHS1), Aldehyde dehydrogenase 2 (ALDH2), 3-hydroxy-3-methylglutaryl-CoA synthase 2 (HMGCS2), Superoxide dismutase 1 (SOD1), Superoxide dismutase 2 (SOD2), Cytochrome P450 family 2 subfamily E member 1 (CYP2E1), Cytochrome P450 family 4 subfamily A member 11 (CYP4A11), Cytochrome P450 family 2 subfamily C member 9 (CYP2C9) and Fatty acid binding protein 1 (FABP-1).
